# Deep exon resequencing of *DLGAP2* as a candidate gene of autism spectrum disorders

**DOI:** 10.1186/2040-2392-4-26

**Published:** 2013-08-01

**Authors:** Wei-Hsien Chien, Susan Shur-Fen Gau, Hsiao-Mei Liao, Yen-Nan Chiu, Yu-Yu Wu, Yu-Shu Huang, Wen-Che Tsai, Ho-Min Tsai, Chia-Hsiang Chen

**Affiliations:** 1 Department of Occupational Therapy, College of Medicine, Fu Jen Catholic University, New Taipei City, Taiwan; 2 Department of Psychiatry, National Taiwan University Hospital and College of Medicine, 7, Chung-Shan South Road, Taipei 10002, Taiwan; 3 Department of Psychology, School of Occupational Therapy, Graduate Institute of Brain and Mind Sciences, and Graduate Institute of Epidemiology and Preventive Medicine, National Taiwan University, Taipei, Taiwan; 4 Department of Psychiatry, Chang Gung Memorial Hospital-Linkou Medical Center, Chang Gung University College of Medicine, Taoyuan, Taiwan; 5 Institute of Medical Sciences, Tzu-Chi University, Hualien, Taiwan

**Keywords:** Autism spectrum disorders, DLGAP2, Common variants, Rare variants

## Abstract

**Background:**

We recently reported a terminal deletion of approximately 2.4 Mb at chromosome 8p23.2-pter in a boy with autism. The deleted region contained the *DLGAP2* gene that encodes the neuronal post-synaptic density protein, discs, large (Drosophila) homolog-associated protein 2. The study aimed to investigate whether *DLGAP2* is genetically associated with autism spectrum disorders (ASD) in general.

**Methods:**

We re-sequenced all the exons of *DLGPA2* in 515 patients with ASD and 596 control subjects from Taiwan. We also conducted bioinformatic analysis and family study of variants identified in this study.

**Results:**

We detected nine common single nucleotide polymorphisms (SNPs) and sixteen novel missense rare variants in this sample. We found that AA homozygotes of rs2906569 (minor allele G, alternate allele A) at intron 1 (*P* = 0.003) and CC homozygotes of rs2301963 (minor allele A, alternate allele C) at exon 3 (*P* = 0.0003) were significantly over-represented in the patient group compared to the controls. We also found no differences in the combined frequency of rare missense variants between the two groups. Some of these rare variants were predicted to have an impact on the function of *DLGAP2* using informatics analysis, and the family study revealed most of the rare missense mutations in patients were inherited from their unaffected parents.

**Conclusions:**

We detected some common and rare genetic variants of *DLGAP2* that might have implication in the pathogenesis of ASD, but they alone may not be sufficient to lead to clinical phenotypes. We suggest that further genetic or environmental factors in affected patients may be present and determine the clinical manifestations.

**Trial registration:**

ClinicalTrial.gov, NCT00494754

## Background

Autism spectrum disorders (ASD) are a group of childhood-onset neurodevelopmental disorders characterized by impaired verbal/nonverbal communication, abnormal reciprocal social interaction, and the presence of stereotyped behaviors and restricted interests. Due to increased awareness and clinical sensitivity to ASD, broadening of the diagnostic criteria of ASD by including Asperger’s disorder and Pervasive Developmental Disorder, not otherwise specified in addition to autistic disorder, and other contributing factors, the prevalence of ASD has increased markedly in the past decade. Recent data showed that up to approximately 11 persons per thousand in the USA are affected with ASD, and males are more predominantly affected than females [1-3]. The literature documents strong evidence of a high degree of genetic influence in the etiology of autism with high heritability estimated to be more than 90% [4]. Genetic approaches such as cytogenetic analysis, genome-wide linkage and association scans, and candidate gene analysis, have been used to dissect the genetic complexity of ASD [5].

Traditional cytogenetic studies [6-9] and the recent array-based comparative genomic hybridization (array CGH) analysis have shown that chromosome abnormalities and rare copy number variants [10,11] are associated with ASD. Various chromosome abnormalities such as deletion, duplication, inversion, and translocation were identified in ASD patients. In particular, the advent of array CGH technology has greatly facilitated the detection of formerly undetectable submicroscopic copy number variants that are associated with ASD [10-14].

Our group previously identified two novel chromosome deletions in ASD using karyotyping analysis and array CGH [15]. One was a terminal deletion of approximately 2.4 Mb at 8p23.2-pter detected in a male patient with autistic disorder [15]. Several genes with neurobiological functions such as discs, large (Drosophila) homolog-associated protein 2 (*DLGAP2*), ceroid-lipofuscinosis, neuronal 8 (*CLN8*), the Rho guanine nucleotide exchange factor 10 (*ARHGEF10*) and F-box protein 25 (*FBXO25*), were mapped to this region. It is likely that haploinsufficiency of one or several of these genes might result in the clinical phenotypes of the affected patients. Hence, these genes might be considered as candidate genes of ASD patients in general.

*DLGAP2* (GeneID 9298) encodes the discs, large (Drosophila) homolog-associated protein 2, which is also called PSD-95/SAP90-binding protein 2 and SAP90/PSD-95-associated protein 2 (SAPAP2). The protein is one of the membrane-associated guanylate kinases localized at the post-synaptic density that plays a role in the molecular organization of synapses and in neuronal cell signaling [16]. These kinases are a family of signaling molecules expressed at various submembrane areas, and contain the PDZ, SH3 and the guanylate kinase domains. Several studies have suggested that the synapse associated proteins (SAPs) localized at postsynaptic density are involved in the pathophysiology of psychiatric disorders [17-21]. Marshall *et al*. detected 13 loci with recurrent CNV in autism cases; they found that several postsynaptic density genes and synapse complex genes were mapped in these CNVs [22]. They also found that a genomic DNA duplication intersected the *DLGAP2* gene in a patient with autism [22]. In addition, Ozgen *et al*. reported a classical inv dup del(8p) in a female patient with autism whose *DLGAP2* gene was located within the 6.9 Mb terminal deletion [23]. Together with the finding in our previous study, these studies suggest that the *DLGAP2* gene is an important candidate gene of ASD. To test this hypothesis, we conducted a deep resequencing of all the exons of the *DLGAP2* in a sample of ASD from Taiwan. Herein, we present our findings of the genetic analysis of *DLGAP2* in this report.

## Methods

### Subjects and procedures

All subjects were Han Chinese from Taiwan. Patients meeting the diagnostic criteria of either autistic disorder or Asperger’s disorder according to the DSM-IV and ICD-10 were enrolled in this study from the psychiatry department of a university hospital, a private medical center, and schools and early intervention centers in northern Taiwan. Subjects with ASD aged 3 to 25 years old, and with a clinical diagnosis of ASD confirmed by the structured interview using the Chinese version of the Autism Diagnostic Interview-Revised (ADI-R) [24,25]. They received clinical assessments and provided DNA data at the university hospital, no matter whether they were recruited from this university hospital or referred by other places. Patients with known chromosomal abnormalities and associated medical conditions were excluded from the study. Subjects receiving routine medical check-ups at the Department of Family Medicine of a medical center were recruited as controls. The mental status of the control subjects was screened by a senior psychiatrist. Individuals with major psychiatric disorders including ASD were excluded.

The Research Ethics Committee of the research sites approved this study. The patients and their parents were informed that participation in this study was completely voluntary and that nonparticipation would not influence their treatment. Written informed consent was obtained from the patients if they were able to understand the contents of the study, the parents of all the patients, and all the control subjects after the procedures were fully explained. A total of 515 patients with ASD were recruited into this study, including 449 male patients (mean age ± standard deviation (SD) = 8.9 ± 4.6 years) and 66 female patients (mean age ± SD = 8.5 ± 4.4 years). The control group comprised 596 individuals including 263 males (mean age ± SD = 42.5 ± 15.1 years) and 333 females (mean age ± SD = 45.2 ± 13.4 years).

The ADI-R data of the 515 patients revealed the scores as 21.19 ± 5.78 in the ‘qualitative abnormalities in reciprocal social interaction’ (cut-off = 10), 15.35 ± 4.19 in the ‘qualitative abnormalities in communication, verbal’ (cut-off = 8), 8.58 ± 3.28 in the ‘qualitative abnormalities in communication, nonverbal’ (cut-off = 7), and 7.14 ± 2.42 in the ‘restricted, repetitive and stereotyped patterns of behaviors’ (cut-off = 3). All patients with ASD were noted to have had abnormal development at or before 36 months.

Genomic DNA was prepared from peripheral blood using the Puregene DNA purification system (Gentra Systems Inc. Minneapolis, MI, USA) according to the manufacturer’s instructions.

### PCR amplification and sequencing

**Figure 1:**
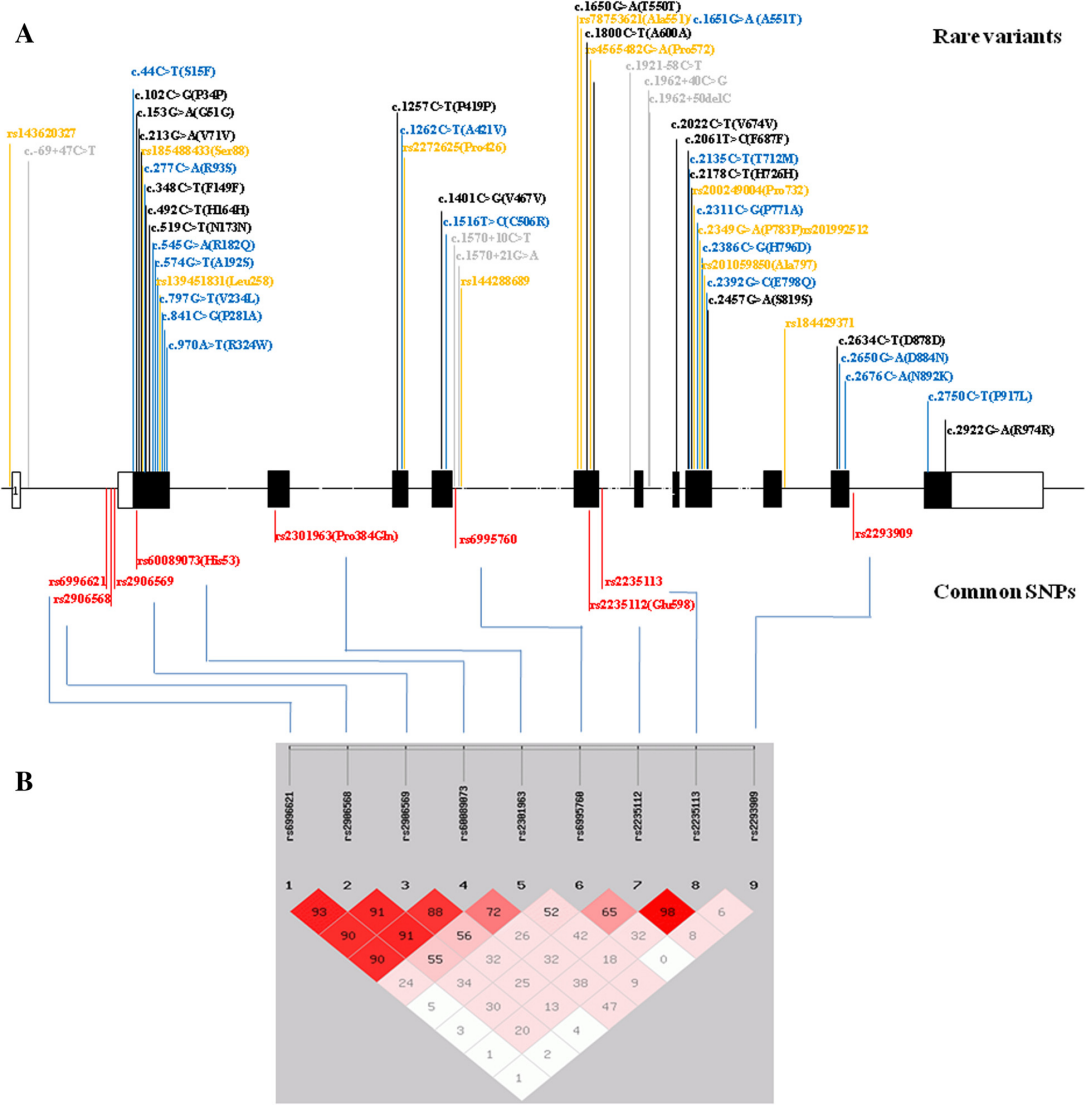
**(A) Schematic genomic structure of the *DLGAP2*, and locations of the common single nucleotide polymorphisms (SNPs) and rare variants identified in this study. (B) **Linkage disequlibrium (LD) analysis of nine common SNPs identified in this study.

The genomic sequences of human *DLGAP2* are available from the NCBI Reference Sequence: NM_004745.3. The human *DLGAP2* comprises twelve exons that span approximately 207 kb on chromosome 8p23.2-23.3 [16]; the schematic genomic structure of the *DLGAP2* is shown in Figure 1. Optimal PCR primer sequences were generated to amplify each exon of the *DLGAP2* using Primer3 (http://bioinfo.ut.ee/primer3/). All the primer sequences, optimal annealing temperatures and size of each amplicon are listed in Additional file 1: Table S1. After PCR, aliquots of PCR products were processed using a PCR Pre-Sequencing Kit (USB, Cleveland, OH, USA) to remove residual primers and dNTPs following the manufacturer’s protocol. The purified PCR products were subjected to direct sequencing using the ABI Prism™ BigDye™ Terminator Cycle Sequencing Ready Reaction Kit Version 3.1, and the ABI autosequencer 3730 (Perkin Elmer Applied Biosystems, Foster City, CA, USA), according to the manufacturer’s protocol. The quality of the sequencing results was visualized using Chromas 2.4.1 software (Technelysium Pty Ltd, South Brisbane, Australia). For variant identification, sequencing results of each subject were aligned and compared with the reference sequences using BioEdit software (http://www.mbio.ncsu.edu/bioedit/bioedit.html). To verify the authenticity of mutations identified in this study, repeated PCR from genomic DNA and re-sequencing of the amplicon in both directions were performed. The nomenclature of genetic variants follows the rules of the ‘Nomenclature for description of human sequence variations’ [26].

### Statistical analysis

For the common single nucleotide polymorphisms (SNPs), the differences in the allele and genotype frequencies between the patients and controls were assessed with the Chi-square test. Fisher's exact test was used to compare the combined frequency of rare variants between the patient and the control groups. It was also used to compare the frequency of damaging and functional rare missense variants between the ASD and control groups. Assessment of haplotype-based association analyses was performed using the SHEsis computer program [27]. A *P* value of less than 0.05 was considered statistically significant, and Bonferroni correction for multiple comparisons was performed when appropriate. We also calculated the *Q* value for each test with the false discovery test of 5% using QVALUE software (http://genomics.princeton.edu/storeylab/qvalue/) [28].

## Results

After sequencing all the exons of *DLGAP2* in 515 patients and 596 control subjects, we identified nine common SNPs (defined as with a minor allele frequency > 0.05), and a total of 49 rare variants (defined as with a frequency < 0.05) in this sample. The locations of these variants are listed in Figure 1.

### Case-control association study of common SNPs

The genotype and allele frequencies of nine common variants in the patients and control subjects are listed in Table 1. Two SNPs (rs2906569 and rs2301963) were found to have significant differences in genotype frequency distribution between the patient and control groups, even after correction for multiple comparisons. The rs2906569 (A > G) was located at intron 1. The genotype AA homozygotes (G: minor allele, A: alternate allele) were significantly over-represented in the patient group compared to the control group (odds ratio: 1.46; 95% confidence interval, 1.13 to 1.87, *P* = 0.003) (Table 2). When the subjects were sub-grouped by gender, the over-representation of genotype AA homozygotes was still observed in male patients but not in female patients (Table 2). The rs2301963 (C > A) was a missense variant (P384Q) located at exon 3 (A: minor allele, C: alternate allele). The CC homozygotes were significantly over-represented in the patient group compared to the control group (odds ratio: 1.30; 95% confidence interval, 0.99 to 1.70; *P* = 0.0003) (Table 2). When the subjects were sub-grouped by gender, the over-representation of CC homozygotes was observed in male patients but not in female patients (Table 2).

**Table 1:** **Allele and genotype frequencies of common SNPs of the *DLGAP2 *gene in ASD patients and control subjects** (**MAF** >**5**%)

**SNP**	**Location**		**Diagnosis**	**n**	**Genotype**			***P *value**	***Q *value**	**Allele**		***P *value**	***Q *value**	**Odds ratio (95 % CI)**
rs6996621	intron 1				C/C	C/A	A/A			C	A			C versus A
c.-68-61C > A		total	autism	454	387 (85.2%)	62 (13.7%)	5 (1.1%)	0.619	0.312	836 (92.1%)	72 (7.9%)	0.401	0.204	1.11 (0.83 to 1.57)
			control	557	467 (83.8%)	80 (14.4%)	10 (1.8%)			1014 (91.0%)	100 (9.0%)			
		male	autism	394	336 (85.3%)	55 (14.0%)	3 (0.7%)	0.593	0.203	727 (92.3%)	61 (7.7%)	0.611	0.611	
			control	246	208 (84.6%)	34 (13.8%)	4 (1.6%)			450 (91.5%)	42 (8.5%)			
		female	autism	60	51 (85%)	7 (11.7%)	2 (3.3%)	0.663	0.266	109 (90.8%)	11 (9.2%)	0.956	0.956	
			control	311	259 (83.3%)	46 (14.8%)	6 (1.9%)			564 (90.7%)	58 (9.3%)			
rs2906568	intron 1				C/C	C/G	G/G			C	G			C versus G
c.-68-25C > G		total	autism	456	188 (41.2%)	169 (37.1%)	99 (21.7%)	0.142	0.112	545 (59.8%)	367 (40.2%)	0.125	0.105	1.15 (0.96 to 1.37)
			control	557	197 (35.4%)	234 (42.0%)	126 (22.6%)			628 (56.4%)	486 (43.6%)			
		male	autism	396	171 (43.2%)	138 (34.8%)	87 (22.0%)	0.072	0.107	480 (60.6%)	312 (39.4%)	0.146	0.299	
			control	246	86 (35.0%)	106 (43.0%)	54(22.0%)			278 (56.5%)	214 (43.5%)			
		female	autism	60	17 (28.3%)	31 (51.7%)	12 (20%)	0.316	0.266	65 (54.2%)	55 (45.8%)	0.671	0.897	
			control	311	111 (35.7%)	128 (41.1%)	72 (23.2%)			350 (56.3%)	272 (43.7%)			
rs2906569	intron 1				A/A	A/G	G/G			A	G			A versus G
c.-68-4A > G		total	autism	458	219 (47.8%)	138 (30.1%)	101 (22.1%)	0.0006	0.095	576 (62.9%)	340 (37.1%)	0.112	0.105	1.16 (0.97 to 1.38)
			control	557	215 (38.6%)	232 (41.7%)	110 (19.7%)			662 (59.4%)	452 (40.6%)			
		male	autism	398	199 (50.0%)	109 (27.4%)	90 (22.6%)	0.0001	0.107	507 (63.7%)	289 (36.3%)	0.075	0.299	
			control	246	91 (37.0%)	107 (43.5%)	48 (19.5%)			289 (58.7%)	203 (41.3%)			
		female	autism	60	20 (33.3%)	29 (48.3%)	11 (18.4%)	0.491	0.266	69 (57.5%)	51 (42.5%)	0.614	0.897	
			control	311	124 (39.9%)	125 (40.2%)	62 (19.9%)			373 (60.0%)	249 (40.0%)			
rs60089073	exon 2				C/C	C/T	T/T			C	T			C versus T
His73		total	autism	466	401 (86.1%)	54 (11.6%)	11 (2.3%)	0.989	0.388	856 (91.8%)	76 (8.2%)	0.891	0.363	0.98 (0.71 to 1.35)
			control	557	481 (86.4%)	63 (11.3%)	13 (2.3%)			1025 (92.0%)	89 (8.0%)			
		male	autism	404	346 (85.6%)	48 (11.9%)	10 (2.5%)	0.236	0.133	740 (91.6%)	68 (8.4%)	0.093	0.299	
			control	246	219 (89.0%)	25 (10.2%)	2 (0.8%)			463 (94.1%)	29 (5.9%)			
		female	autism	62	55 (88.7%)	6 (9.7%)	1 (1.6%)	0.607	0.266	116 (93.5%)	8 (6.5%)	0.259	0.897	
			control	311	262 (84.3%)	38 (12.2%)	11 (3.5%)			562 (90.4%)	60 (9.6%)			
rs2301963	exon 3				C/C	C/A	A/A			C	A			C versus A
Pro384Gln		total	autism	513	171 (33.3%)	231 (45.1%)	111 (21.6%)	0.0005	0.095	573 (55.8%)	453 (44.2%)	0.026	0.105	1.21 (1.02 to 1.43)
			control	593	139 (23.4%)	328 (55.%)	126 (21.2%)			606 (51.1%)	580 (48.9%)			
		male	autism	448	150 (33.5%)	202 (45.1%)	96 (21.4%)	0.068	0.107	502 (56.0%)	394 (44.0%)	0.102	0.299	
			control	260	66 (25.4%)	136 (52.3%)	58 (22.3%)			268 (51.5%)	252 (48.5%)			
		female	autism	65	21 (32.3%)	29 (44.6%)	15 (23.1%)	0.124	0.266	71 (54.6%)	59 (45.4%)	0.420	0.897	
			control	333	73 (21.9%)	192 (57.7%)	68 (20.4%)			338 (50.8%)	328 (49.2%)			
rs6995760	intron 5				A/A	A/G	G/G			A	G			A versus G
c.1570 + 14A > G		total	autism	500	346 (69.2%)	141 (28.2%)	13 (2.6%)	0.913	0.388	833 (83.3%)	167 (16.7%)	0.925	0.363	1.01 (0.80 to 1.27)
			control	531	368 (69.3%)	147 (27.7%)	16 (3.0%)			883 (83.1%)	179 (16.9%)			
		male	autism	432	305 (70.6%)	115 (26.6%)	12 (2.8%)	0.502	0.193	725 (83.9%)	139 (16.1%)	0.245	0.299	
			control	234	155 (66.3%)	71 (30.3%)	8 (3.4%)			381 (81.4%)	87 (18.6%)			
		female	autism	68	41 (60.3%)	26 (38.2%)	1 (1.5%)	0.103	0.266	108 (79.4%)	28 (20.6%)	0.148	0.897	
			control	297	213 (71.7%)	76 (25.6%)	8 (2.7%)			502 (84.5%)	92 (15.5%)			
rs2235112	exon 6				A/A	A/G	G/G			A	G			A versus G
Glu598		total	autism	509	95 (18.8%)	233 (45.7%)	181 (35.5%)	0.028	0.095	424 (41.6%)	595 (58.4%)	0.012	0.105	1.29 (1.05 to 1.50)
			control	513	66 (12.9%)	239 (46.6%)	208 (40.5%)			371 (36.2%)	655 (63.8%)			
		male	autism	444	85 (19.1%)	205 (46.2%)	154 (34.7%)	0.049	0.107	375 (42.2%)	513 (57.8%)	0.014	0.299	
			control	224	28 (12.5%)	102 (45.5%)	94 (42.0%)			158 (35.3%)	290 (64.7%)			
		female	autism	66	11 (16.7%)	28 (42.4%)	27 (40.9%)	0.671	0.266	50 (37.9%)	82 (62.1%)	0.825	0.928	
			control	289	38 (13.2%)	137 (47.4%)	114 (39.4%)			213 (36.9%)	365 (63.1%)			
rs2235113	intron 6				C/C	C/G	G/G			C	G			C versus G
c.1920 + 19C > G		total	autism	510	156 (30.6%)	233 (45.7%)	121 (23.7%)	0.050	0.095	545 (53.4%)	475 (46.6%)	0.030	0.105	1.21 (1.02 to 1.44)
			control	509	121 (23.8%)	253 (49.7%)	135 (26.5%)			495 (48.6%)	523 (51.4%)			
		male	autism	443	137 (30.9%)	205 (46.3%)	101 (22.8%)	0.303	0.139	479 (54.1%)	407 (45.9%)	0.243	0.299	
			control	222	56 (25.2%)	113 (50.9%)	53 (23.9%)			225 (50.7%)	219 (49.3%)			
		female	autism	67	19 (28.3%)	28 (41.8%)	20 (29.9%)	0.513	0.266	66 (49.3%)	68 (50.7%)	0.644	0.897	
			control	287	65 (22.6%)	140 (48.8%)	82 (28.6%)			270 (47.0%)	304 (53.0%)			
rs2293909	intron 11				T/T	T/C	C/C			T	C			T versus C
c.2709 + 71T > C		total	autism	460	255 (55.4%)	166 (36.1%)	39 (8.5%)	0.012	0.095	676 (73.5%)	244 (26.5%)	0.012	0.105	1.28 (1.06 to 1.55)
			control	573	265 (46.2%)	254 (44.3%)	54 (9.4%)			784 (68.4%)	362 (31.6%)			
		male	autism	403	230 (57.1%)	138 (34.2%)	35 (8.7%)	0.117	0.107	598 (74.2%)	208 (25.8%)	0.165	0.299	
			control	256	127 (49.6%)	108 (42.2%)	21 (8.2%)			362 (70.7%)	150 (29.3%)			
		female	autism	57	25 (43.9%)	28 (49.1%)	4 (7.0%)	0.719	0.266	78 (68.4%)	36 (31.6%)	0.698	0.897	
			control	317	138 (43.5%)	146 (46.1%)	33 (10.4%)			422 (66.6%)	212 (33.4%)			

ASD, autistic spectrum disorders; MAF, minor allele frequency; SNPs, single nucleotide polymorphisms.

**Table 2:** **Odds ratio of rs2906569 and rs2301963 of the *DLGAP2 *gene in patients with autism and control subjects**

**SNP**	**Location**		**Diagnosis**	**n**	**Odds ratio (95% CI)**	***P *value**
rs2906569	intron 1	total			A/A versus A/G + G/G	
			autism	458	1.46 (1.13 to1.87)	0.003^a^
			control	557		
		male	autism	398	1.70 (1.23 to 2.36)	0.001^a^
			control	246		
		female	autism	60	0.75 (0.42 to 1.35)	0.341
			control	311		
rs2301963	exon 3	total			C/C versus C/A + A/A	*P* value
			autism	513	1.63 (1.25 to 2.13)	0.0003^a^
			control	593		
		male	autism	448	1.48 (1.05 to 2.08)	0.024^a^
			control	260		
		female	autism	65	1.70 (0.95 to 3.04)	0.071
			control	333		

^a^*P* value < 0.05; SNP, single nucleotide polymorphism.

Further linkage disequilibrium (LD) analysis showed strong LD among rs6996621, rs2906568, rs2906569, and rs60089073. In addition, rs2235112 and rs2235113 also showed strong LD (Figure 1). In haplotype-based association analysis derived from nine SNPs, we found significant difference in the haplotype distribution of ACACAAGGT and CCACCAACT between the ASD patients and controls, but only haplotype CCACCAACT was sustained after correction for multiple comparisons (Table 3).

**Table 3:** The distributions of haplotypes in ASD and controls

	**Haplotype**	**Cases (frequency)**	**Controls (frequency)**	**Chi**^**2**^	**Fisher's *P***	**Pearson's *P***	**Odds ratio (95%CI)**
1	A C A C A A G G T	8.84 (0.012)	28.20 (0.036)	7.081	0.007814^a^	0.007806^a^	0.367 (0.170 to 0.790)
2	C C A C C A A C C	41.17 (0.057)	63.13 (0.080)	1.548	0.213443	0.213365	0.768 (0.506 to 1.165)
3	C C A C C A A C T	84.15 (0.117)	60.19 (0.076)	11.981	0.000542^a^	0.000541^a^	1.880 (1.310 to 2.698)
4	C C A C C A G G C	12.99 (0.018)	26.04 (0.033)	2.331	0.126865	0.126793	0.592 (0.300 to 1.168)
5	C C A C C A G G T	86.66 (0.121)	89.80 (0.114)	1.380	0.240147	0.240071	1.218 (0.876 to 1.694)
6	C C A T C A A C T	27.74 (0.039)	25.95 (0.033)	0.973	0.324045	0.323985	1.319 (0.759 to 2.292)
7	C G G C A A A C T	31.96 (0.045)	32.97 (0.042)	0.465	0.495147	0.495131	1.192 (0.719 to 1.976)
8	C G G C A A G G C	30.14 (0.042)	45.29 (0.057)	0.902	0.342270	0.342214	0.792 (0.490 to 1.282)
9	C G G C A A G G T	60.91 (0.085)	81.38 (0.103)	0.381	0.537097	0.537090	0.892 (0.622 to 1.281)
10	C G G C A G G C T	19.59 (0.027)	37.66 (0.048)	2.960	0.085418	0.085359	0.613 (0.349 to 1.076)

ASD, autistic spectrum disorders; Global result: total control = 788.0, total case = 718.0; global chi^2^ is 26.804893 while df = 9 (frequency < 0.03 in both control and case has been dropped). Fisher's *P* value is 0.001537. Pearson's *P* value is 0.001507. Note: ^a^P ≤ value 0.05.

### Identification of rare genetic variants of *DLGAP2*

In this study, we found a total of 16 rare missense mutations in our ASD patients and control subjects. The locations of these variants are illustrated in Figure 1. These missense variants are novel and have not been reported before in the literature. Distributions of these rare missense variants are listed in Table 4. There was no significant difference in the combined frequency of rare missense mutations between the two groups (*P* = 1.00). Those individuals who carried these rare missense mutations had only one missense mutation; we found no individual who carried two rare missense variants simultaneously.

**Table 4:** **Unique nonsynonymous variants of the *DLGAP2 *gene identified in ASD patients and controls and their functional predictions**

**Location**			***In silico *analysis**					
	**Nucleotide position**	**Variants**	**PolyPhen**-**2**	**SIFT**	**Autism**	**Control**	**rs2906569**	**rs2301963**
exon2	c.44 C > T	S15F	probably damaging	affect protein function	U1974		A/A	C/A
	c.277 C > A	R93S	probably damaging	tolerated	U1843		A/G	C/A
	c.545 G > A	R182Q	probably damaging	tolerated	U173		A/G	C/A
	c.574 G > T	A192S	benign	tolerated	U396		A/A	C/C
	c.797 G > T	V234L	benign	tolerated		ZN4215	G/G	C/C
	c.797 G > T	V234L	benign	tolerated		HN616	A/A	C/A
	c.841 C > G	P281A	probably damaging	tolerated	U323		A/G	C/A
	c.841 C > G	P281A	probably damaging	tolerated	U1519		G/G	A/A
	c.841 C > G	P281A	probably damaging	tolerated	U1988		G/G	C/A
	c.841 C > G	P281A	probably damaging	tolerated		ZN4014	A/G	missing
	c.841 C > G	P281A	probably damaging	tolerated		HN449	A/G	C/A
	c.841 C > G	P281A	probably damaging	tolerated		HN581	A/G	C/A
	c.970 A > T	R324W	probably damaging	affect protein function	U1803		A/A	C/A
exon4	c.1262 C > T	A421V	benign	tolerated		ZN4205	G/G	A/A
exon5	c.1516 T > C	C506R	probably damaging	affect protein function	U1247		missing	C/A
exon9	c.2135 C > T	T712M	benign	tolerated	U2096		G/G	C/A
	c.2135 C > T	T712M	benign	tolerated		HN576	missing	C/A
	c.2135 C > T	T712M	benign	tolerated		HN278	A/A	C/A
	c.2311 C > G	P771A	benign	tolerated		ZN4182	A/A	C/C
	c.2386 C > G	H796D	probably damaging	affect protein function		ZN4053	A/G	C/A
	c.2392 G > C	E798Q	probably damaging	affect protein function	U1082		A/A	C/C
exon11	c.2650 G > A	D884N	benign	tolerated		HN526	G/G	C/A
	c.2676 C > A	N892K	possibly damaging	affect protein function		ZN4107	missing	missing
exon12	c.2750 C > T	P917L	benign	tolerated	U1000		A/G	C/A
	c.2750 C > T	P917L	benign (0.002)	tolerated	U2098		G/G	C/A
	c.2750 C > T	P917L	benign (0.002)	tolerated		HN444	A/A	C/C
PolyPhen-2	autism	control	*P* (Fisher’s test)	SIFT	autism	control	*P* (Fisher’s test)	
damaging	9	5	0.12	functional	4	2	0.32	
benign	4	8		tolerated	9	11		

### Family study and functional prediction of rare variants

**Figure 2:**
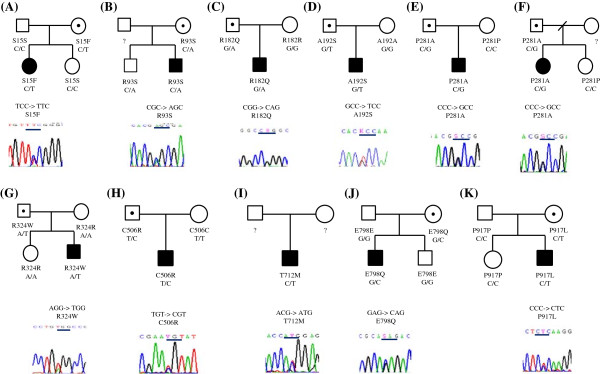
**(A) Pedigree of S15F mutation, (B) Pedigree of R93S mutation, (C) Pedigree of R182Q mutation. (D) **Pedigree of A192S mutation. **(E,F)** Pedigrees of P281A mutation. **(G)** Pedigree of R324W mutation. **(H)** Pedigree of C506R mutation. **(I)** Pedigree of T712M mutation. **(J)** Pedigree of E798Q mutation. **(K)** Pedigree of P917L mutation.

A total of 10 missense variants (S15F, R93S, R182Q, A192S, P281A, R324W, C506R, T712M, E798Q and P917L) were detected in 13 patients (Table 4). All the patients carrying these rare variants were heterozygotes. Four of the 13 patients (A, B, J, K) inherited the variant from their mothers and 6 (C, D, E, F, G, H) from their fathers (Figure 2). Three patients did not have enough genetic information from the parents. Among these ten missense variants, seven (S15F, R93S, R182Q, A192S, R324W, C506R and E798Q were found in the patient group only, while the other three (P281A, T712M and P917L) were detected also in the control group. Aside from these three variants that overlapped with the patient group, six missense variants (V234L, A421V, P771A, H796D, D884N, and N892K) were unique in the control group. The inheritance mode of missense variants found in the control group cannot be traced because we did not collect their parents’ DNA. In the analysis of functional prediction of these 16 rare missense variants, S15F, R93S, R182Q, P281A, R324W, C506R, H796D, E798Q, and N892K were predicted to have functional impact on the protein using the PolyPhen-2 or SIFT computer program (Table 4).

The ADI-R data of these 13 patients revealed scores of 22.82 ± 7.15 (range, 8 to 28) in the ‘qualitative abnormalities in reciprocal social interaction’ (cut-off = 10), 15.00 ± 4.84 (range, 5 to 22) in the ‘qualitative abnormalities in communication, verbal’ (cut-off = 8), 9.55 ± 4.39 (range, 4 to 14) in the ‘qualitative abnormalities in communication, nonverbal’ (cut-off = 7), and 6.00 ± 2.68 (range, 1 to 12) in the ‘restricted, repetitive and stereotyped patterns of behaviors’ (cut-off = 3). The average age at which the 13 patients said their first word was 37.1 ± 17.4 (range, 15 to 66) months old and at which they said their first phrase was 44.8 ± 18.5 (range, 19 to 78) months. Their current average intelligence quotients (IQ) were 82.8 ± 34.0 (range 40 to 126) for full-scale IQ, 84.9 ± 32.1 (range, 41 to 123) for performance IQ, and 82.3 ± 33.1 (range, 44 to 122 ) for verbal IQ, as assessed by the Wechsler Intelligence Scale for Children-third edition. Due to the lack of psychometric data for the control subjects who carried the rare missense mutations, we were not able to determine their clinical characteristics.

Clinical assessments of the parents of the 10 patients carrying rare missense mutations found that none of them achieved the clinical diagnosis of ASD. Their reports on the Chinese version of the Autism Spectrum Quotient [29] also revealed no evidence of an overt autistic trait.

## Discussion

In this study, we found that rs2906569 at intron 1 and rs2301963 (P384Q) at exon 3 of *DLGAP2* were associated with ASD. These two SNPs did not form significant LD in our genetic analysis. The functional significance of rs2906569 was difficult to infer, because it was located at intron 1. As to rs2301963 (C > A, P384Q), the A allele (Q allele) was the minor allele in our population, and was predicted to be probably damaging using the PolyPhen-2 computer program, but tolerated by SIFT. Based on the finding of significant over-representation of the CC homozygotes in the patient group, we suggest that the Q384 variant of the DLGAP2 might confer an increased risk of ASD, but the real mechanism and meaning of this association remain to be clarified. The small sample size of this study may have led to a false positive, which is also a limitation of this study. In a recent review of the role of common variants in autism, Devlin and colleagues reported their study on three large genome-wide association (GWA) studies of autism, each of which showed a single, non-overlapping risk locus. They found that there was no significant finding when all the data were combined. In their analysis, they found no definitive, replicated results, and they could not be certain that there was a role for common variants in autism risk [30]. Hence, an independent replication study is needed to verify our finding.

We also detected a total of 16 novel missense rare variants in the patient and control groups in this study. Given that some of these missense mutations were predicted to have functional impact on *DLGAP2*, we found no differences in their combined frequency between the two groups. In addition, most of these rare missense mutations found in the patients were inherited from their unaffected parents. Hence, the clinical relevance of these rare missense mutations to ASD is not straightforward, and needs further elucidation.

We attempted to assess the interactions between rs2906569 and rs2301963 and the missense rare variants in this study, but could not detect interactions between them. The small sample size of this study might be a major limiting factor. There were eight AA homozygotes of rs2906569 carrying rare missense variants, including four patients and four controls, and there were five CC homozygotes of rs2301963 carrying rare missense variants, including two patients and three controls. The genotype of rs2906569 and rs2301963 in subjects carrying the rare missense variants are listed in the Table 4.

To assess the phenotypic significance of two common SNPs associated with ASD, we compared the three core symptoms of ASD measured by the ADI-R and the Chinese version of Social Communication Questionnaire (SCQ) between patients with A/A and patients with A/G + G/G of rs2906569 (Additional file 2: Table S2), between patients with C/C and patients with C/A + A/A of rs2301963 (Additional file 3: Table S3). We also compared the three core symptoms of ASD measured by the ADI-R and SCQ between patients who carried rare missense mutations and those did not carry rare missense mutations (Additional file 4: Table S4). The Chinese SCQ is a screening tool based on the Autism Diagnostic Interview-Revised (ADI-R) algorithm which corresponds to DSM-IV diagnostic criteria. It examines the three functional domains of reciprocal social interaction, communication, and restricted, repetitive, and stereotyped patterns. The Chinese SCQ was translated under the approval of Western Psychological Services and was validated by the research group led by Gau and Wu [31]. The results showed that there were no statistical significant differences in all the comparisons (all *P* values > 0.1), regardless of gender with the following two exceptions. Patients with A/A of rs2906569 had less severe social interaction impairment than patients with A/G + G/G of rs2906569 (*P* = 0.0219) and such significant difference was only noted in male patients (*P* = 0.0285). But, the statistical significance did not sustain after correcting for multiple testing.

ASD is a complex disease with highly heterogeneous genetic underpinnings, and genotype-phenotype correlation remains a challenging task. According to the common variant hypothesis of complex psychiatric disorders, the effect size of common variants is usually considered small or modest. Moreover, it is not unusual to find inconsistent results among different studies, and the common variants can only explain a small proportion of the clinical variance. In a recent report on stage two of the autism genome project genome-wide association study, Anney and colleagues found that no single SNP showed a significant association with ASD or selected phenotypes at a genome-wide level after genotyping over a million SNPs, and the clinical variance explained by common variants *en masse* was small [32].

In contrast, the ‘rare variant hypothesis’ of complex psychiatric disorders suggests that rare variants are likely to have large effect sizes and to be *de novo* in their origin [33]. Given that several studies have provided evidence to support the large effect size of *de novo* rare mutations associated with ASD [34-36], emerging evidence suggests the multiple-hits model may be more appropriate to explain the incomplete penetrance and varied expressivity of genetic underpinnings of ASD [37,38]. Girirajan and colleagues proposed a ‘two-hit’ model in which a first hit may act in concert with some factors as a second hit, such as mutations in a single gene, micro-deletions/duplications, epigenetic factors, or environmental insults, and result in variable expressivity of phenotypes in complex neuropsychiatric disorders [38]. Furthermore, in a genetic study of high-functioning, idiopathic ASD, Schaaf and colleagues reported that in addition to *de novo* rare mutations, patients with ASD had a significantly higher proportion of multiple events of oligogenic heterozygosity than control subjects, suggesting oligogenic heterozygosity is a new potential mechanism in the pathogenesis of ASD [39]. In our previous study, we also reported a boy with ASD who carried two CNVs that were inherited respectively from his unaffected parents [40]. Our previous case report also lent support to the two-hit and compound heterozygosity models of ASD.

In a recent study examining the patterns and rates of exonic *de novo* mutations in ASD, Neale and colleagues found only a small increase in the rate of *de novo* events in ASD. They suggested an important but limited role for *de novo* point mutations in ASD, and supported polygenic models of ASD [41]. In the present study, we found that the putative deleterious missense variants occurred in both patient and control groups with equal chance, and that most of the rare missense mutations were inherited from their unaffected parents. We suggest that the missense mutations of *DLGAP2* alone may not be sufficient for the clinical presentations of ASD, and additional hits such as environmental insults or further genetic mutations in the affected patients may be needed for the clinical presentations, which support ASD as likely a multifactorial disease. Hence, it is difficult to find a strong association with a single gene, like *DLGAP2* in this study.

## Conclusions

We identified two common SNPs and some rare missense mutations of *DLGAP2* that might be implicated in the pathogenesis of ASD. However, their clinical relevance is not straightforward. We suggest additional genetic or environmental factors in the affected patients might be present to determine the clinical presentations. The findings from this study can only be considered as preliminary as it has only a limited sample size. Further independent replication studies are needed to verify our findings in the present study.

## Abbreviations

ADI-R: Autism diagnostic interview-revised; ARHGEF10: Rho guanine nucleotide exchange factor 10; Array CGH: Array-based comparative genomic hybridization; ASD: Autism spectrum disorders; CI: Confidence interval; CLN8: Ceroid-lipofuscinosis, neuronal 8; DSM-IV: Diagnostic and statistical manual of mental disorders, fourth edition; DLGAP2: Disc, large (Drosophila) homolog-associated protein 2; FBXO25: F-box protein 25; GWA: Genome-wide association; IQ: Intelligence quotient; LD: Linkage disequilibrium; MAF: Minor allele frequency; PCR: Polymerase chain reaction; SAPAP2: SAP90/PSD-95-associated protein 2; SAPs: Synapse associated proteins; SNP: Single nucleotide polymorphism; SD: Standard deviation.

## Competing interests

The authors declare that they do not have competing interests.

## Authors’ contributions

The contributions of the authors are as follows: SSG was the principle investigator in this project. SSG and CHC designed the study and wrote the protocol. SSG trained the clinical research team, supervised in research execution, and collected all the clinical data of the ASD cases. SSG and YYW were responsible for the ADI-R training and interviews. SSG, YYW, WCT, YNC, and YSH were responsible for clinical diagnoses and helped recruit the patients (in the order of number of subjects recruited) and CHC screened for mental disorders in the controls. WHC, HML and HMT performed the experimental works and analyzed the data. WHC drafted the manuscript. CHC and SSG critically revised the manuscript and all the authors approved the manuscript.

## Web resources

Primer3: http://bioinfo.ut.ee/primer3/. SHEsis: http://analysis2.bio-x.cn/myAnalysis.php. PolyPhen-2: http://genetics.bwh.harvard.edu/pph2/. SIFT: http://sift.jcvi.org/.

## Supplementary Material

Additional file 1: Table S1 Primer sequences, optimal annealing temperature (Ta) and size of PCR products of the DLGAP2 gene. Click here for file

Additional file 2: Table S2 Comparison of the three core symptoms of autism spectrum disorder measured by the ADI-R and SCQ between patients with A/A versus patients with A/G + G/G of rs2906569 stratified by gender. Click here for file

Additional file 3: Table S3 Comparison of the three core symptoms of autism spectrum disorder measured by the ADI-R and SCQ between patients with C/C versus patients with C/A + A/A of rs2301963 stratified by gender. Click here for file

Additional file 4: Table S4. Comparison of the three core symptoms of autism spectrum disorder measured by the ADI-R and SCQ between 11 patients who were found to have rare missense mutations with the rest of the patients. Click here for file
