# Supplementary material for: Deep exon resequencing of DLGAP2 as a candidate gene of autism spectrum disorders
Source: Mol Autism. 2013 Aug 1;4:26. doi: 10.1186/2040-2392-4-26 (PMC3751063; doi:10.1186/2040-2392-4-26)
Supplement: Additional file 1: Table S1 — Primer sequences, optimal annealing temperature (Ta) and size of PCR products of the DLGAP2 gene. [file 2040-2392-4-26-S1.doc]

# Supplementary Table 1 Primer sequences, optimal annealing temperature (Ta) and size of PCR products of the DLGAP2 gene.

| **Amplicon** | **Forward (5’-3’)** | **Reverse (5’-3’)** | **Ta (℃)** | **Size (bp)** |
| --- | --- | --- | --- | --- |
| Exon 1 | tgaagatgtgcagggaatga | gctaacgtgtgtttgtggga | 60 | 242 |
| Exon 2.1 | aacccacaaatctgccctct | gaagagcttctgcacggagt | 63 | 691 |
| Exon 2.2 | acgctgcagtaccagaggac | atgcagttgttatctgtcaattaaa | 58 | 697 |
| Exon 3 | tccaaaaaggagctgatgct | cgcaggcagtgggaaagt | 63 | 334 |
| Exon 4 | gtgtgggttggatggtcatt | agactggaccccagggag | 65 | 269 |
| Exon 5 | gtaacgtgatggtgaccctg | tatgcctctagagtccccgc | 63 | 326 |
| Exon 6 | agtagaccacaggctgacgg | tgctggggttacagtcagtg | 63 | 529 |
| Exon 7 | gagtggagcgtgctgagag | tgcctggcacatagaatcag | 60 | 293 |
| Exon 8 | caaaatagtcccttgcccag | aaggacaggcatgattgagg | 63 | 257 |
| Exon 9 | tcctctcagaagggctacca | cacttgaataccaaagggagga | 60 | 570 |
| Exon 10 | gctcccttggtgtgatgttt | aatgatgcccattagcttgg | 60 | 235 |
| Exon 11 | ttctccctaatccgcctctt | gggaaatttgctcgtgtgtt | 58 | 388 |
| Exon 12 | ttccacaaatcccatcctgt | gggaggaggagacagaaacc | 60 | 514 |
