# Supplementary material for: Deep exon resequencing of DLGAP2 as a candidate gene of autism spectrum disorders
Source: Mol Autism. 2013 Aug 1;4:26. doi: 10.1186/2040-2392-4-26 (PMC3751063; doi:10.1186/2040-2392-4-26)
Supplement: Additional file 2: Table S2 — Comparison of the three core symptoms of autism spectrum disorder measured by the ADI-R and SCQ between patients with A/A versus patients with A/G + G/G of rs2906569 stratified by gender. [file 2040-2392-4-26-S2.docx]

**Supplementary Table 2 Comparison of the three core symptoms of autism spectrum disorder measured by the ADI-R and SCQ between patients with A/A vs patients with A/G+G/G of rs2906569 stratified by gender**

|  | Total | | | Male | | | Female | | |
| --- | --- | --- | --- | --- | --- | --- | --- | --- | --- |
|  | A/A | A/G+G/G | F value  (p value) | A/A | A/G+G/G | F value  (p value) | A/A | A/G+G/G | F value  (p value) |
|  | N=178(219) | N=186(239) |  | N=162 | N=158 |  | N=16 | N=28 |  |
| ADI-R-SOC | 21.03±5.81 | 21.41±5.71 | 0.40  (0.5291) | 21.12±5.76 | 21.27±5.53 | 0.06  (0.8064) | 20.13±6.42 | 22.18±6.73 | 0.98  (0.3278) |
| ADI-R COM | 15.39±4.28 | 15.28±4.14 | 0.06  (0.8063) | 15.4±4.31 | 15.29±4.3 | 0.05  (0.8191) | 15.31±4.14 | 15.25±3.22 | 0.00  (0.9558) |
| ADI-R BEV | 7.14±2.35 | 7.15±2.58 | 0.00  (0.9855) | 7.27±2.29 | 7.35±2.54 | 0.11  (0.7419) | 5.88±2.68 | 5.96±2.55 | 0.01  (0.9131) |
| SCQ-SOC | 10.62±5.41 | 11.96±5.46 | 5.30  (0.0219) | 10.57±5.44 | 11.94±5.52 | 4.84  (0.0285) | 11.69±4.92 | 11.84±5.26 | 0.01  (0.9280) |
| SCQ -BEV | 5.18±2.67 | 5.07±2.69 | 0.15  (0.6946) | 5.29±2.65 | 5.11±2.63 | 0.38  (0.5383) | 4.06±2.77 | 4.73±2.99 | 0.52  (0.4736) |
| SCQ -COM | 4.21±1.98 | 3.96±2.03 | 1.39  (0.2385) | 4.23±2 | 3.99±2.03 | 1.11  (0.2924) | 3.94±1.81 | 3.85±2.13 | 0.02  (0.8872) |
| SCQ-Total | 18.69±7.06 | 19.84±6.79 | 2.42  (0.1205) | 18.71±7.18 | 19.92±6.82 | 2.27  (0.1333) | 18.81±6 | 19.17±6.77 | 0.03  (0.8644) |

Note: ADI-R = Autism Diagnostic Interview-Revised; SCQ = Social Communication Questionnaire; ADI-R-SOC = Qualitative Abnormalities in Reciprocal Social Interaction of the ADI-R; ADI-R COM = Qualitative Abnormalities in Communication (Verbal) of the ADI-R; ADI-R BEV = Restricted, Repetitive, and Stereotyped Patterns of Behavior of the ADI-R; SCQ-SOC = Social Interaction Subscale of the SCQ ; SCQ –BEV = Repetitive Behavior Subscale; SCQ-COM = Communication Subscale; SCQ-Total = total score of the SCQ
