# Supplementary material for: Deep exon resequencing of DLGAP2 as a candidate gene of autism spectrum disorders
Source: Mol Autism. 2013 Aug 1;4:26. doi: 10.1186/2040-2392-4-26 (PMC3751063; doi:10.1186/2040-2392-4-26)
Supplement: Additional file 3: Table S3 — Comparison of the three core symptoms of autism spectrum disorder measured by the ADI-R and SCQ between patients with C/C versus patients with C/A + A/A of rs2301963 stratified by gender. [file 2040-2392-4-26-S3.docx]

**Supplementary Table 3 Comparison of the three core symptoms of autism spectrum disorder measured by the ADI-R and SCQ between patients with C/C vs patients with C/A+A/A of rs2301963 stratified by gender**

|  | Total | | | Male | | | Female | | |
| --- | --- | --- | --- | --- | --- | --- | --- | --- | --- |
|  | A/A | A/G+G/G | F value  (p value) | A/A | A/G+G/G | F value  (p value) | A/A | A/G+G/G | F value  (p value) |
|  | N=137(171 | N=266(342) |  | N=115 | N=235 |  | N=14 | N=31 |  |
| ADI-R-SOC | 21.34±5.33 | 21.09±5.95 | 0.17  (0.6847) | 21.37±5.44 | 20.93±5.81 | 0.49  (0.4853) | 21±4.47 | 22.29±6.88 | 0.41  (0.5249) |
| ADI-R COM | 15.49±4.19 | 15.24±4.17 | 0.32  (0.5718) | 15.54±4.3 | 15.19±4.22 | 0.53  (0.4655) | 15.07±3.25 | 15.61±3.79 | 0.21  (0.6460) |
| ADI-R BEV | 7.13±2.47 | 7.13±2.46 | 0.00  (0.9890) | 7.25±2.39 | 7.29±2.44 | 0.02  (0.9024) | 6.07±3 | 5.94±2.31 | 0.03  (0.8686) |
| SCQ-SOC | 10.93±5.45 | 11.52±5.72 | 0.94  (0.3317) | 10.99±5.6 | 11.38±5.7 | 0.37  (0.5448) | 10.38±4.01 | 12.69±5.64 | 1.77  (0.1902) |
| SCQ -BEV | 5.27±2.7 | 5.04±2.66 | 0.59  (0.4410) | 5.32±2.61 | 5.14±2.65 | 0.36  (0.5500) | 4.77±3.47 | 4.13±2.58 | 0.45  (0.5084) |
| SCQ -COM | 4.21±1.95 | 4.07±2.05 | 0.40  (0.5275) | 4.29±1.95 | 4.07±2.04 | 0.85  (0.3568) | 3.54±1.94 | 4.03±2.14 | 0.51  (0.4787) |
| SCQ-Total | 19.1±6.6 | 19.39±7.25 | 0.15  (0.6991) | 19.28±6.77 | 19.35±7.25 | 0.01  (0.9308) | 17.54±4.79 | 19.74±7.36 | 0.98  (0.3280) |

Note: ADI-R = Autism Diagnostic Interview-Revised; SCQ = Social Communication Questionnaire; ADI-R-SOC = Qualitative Abnormalities in Reciprocal Social Interaction of the ADI-R; ADI-R COM = Qualitative Abnormalities in Communication (Verbal) of the ADI-R; ADI-R BEV = Restricted, Repetitive, and Stereotyped Patterns of Behavior of the ADI-R; SCQ-SOC = Social Interaction Subscale of the SCQ ; SCQ –BEV = Repetitive Behavior Subscale; SCQ-COM = Communication Subscale; SCQ-Total = total score of the SCQ
