# Supplementary material for: Deep exon resequencing of DLGAP2 as a candidate gene of autism spectrum disorders
Source: Mol Autism. 2013 Aug 1;4:26. doi: 10.1186/2040-2392-4-26 (PMC3751063; doi:10.1186/2040-2392-4-26)
Supplement: Additional file 4: Table S4. — Comparison of the three core symptoms of autism spectrum disorder measured by the ADI-R and SCQ between 11 patients who were found to have rare missense mutations with the rest of the patients. [file 2040-2392-4-26-S4.docx]

**Supplementary Table 4 Comparison of the three core symptoms of autism spectrum disorder measured by the ADI-R and SCQ between 11 patients who were found to have rare missense mutations with the rest of the patients.**

|  | Total | | | Male | | | Female | | |
| --- | --- | --- | --- | --- | --- | --- | --- | --- | --- |
|  | Yes* | None | F value  (p value) | Yes* | None | F value  (p value) | Yes* | None | F value  (p value) |
|  | N=11(13) | N=394(502) |  | N=9 | N=351 |  | N=2 | N=43 |  |
| ADI-R-SOC | 22.82±7.15 | 21.09±5.69 | 0.97  (0.3244) | 22±7.7 | 21.02±5.62 | 0.26  (0.6085) | 26.5±2.12 | 21.67±6.26 | 1.16  (0.2878) |
| ADI-R COM | 15±4.84 | 15.29±4.16 | 0.05  (0.8209) | 14.78±5.29 | 15.27±4.22 | 0.12  (0.7298) | 16±2.83 | 15.42±3.66 | 0.05  (0.8264) |
| ADI-R BEV | 6±2.68 | 7.14±2.43 | 2.34  (0.1266) | 6.67±2.45 | 7.27±2.4 | 0.55  (0.4593) | 3±1.41 | 6.12±2.47 | 3.09  (0.0860) |
| SCQ-SOC | 13.4±6.69 | 11.27±5.62 | 1.39  (0.2387) | 12.25±7.07 | 11.22±5.66 | 0.25  (0.6142) | 18±0 | 11.7±5.21 | 2.85  (0.0988) |
| SCQ -BEV | 4.1±2.88 | 5.12±2.64 | 1.45  (0.2295) | 4.5±3.12 | 5.19±2.6 | 0.55  (0.4591) | 2.5±0.71 | 4.41±2.89 | 0.86  (0.3600) |
| SCQ -COM | 3.5±1.18 | 4.13±2.02 | 0.96  (0.3281) | 3.88±0.99 | 4.15±2.02 | 0.14  (0.7062) | 2±0 | 3.98±2.08 | 1.76  (0.1914) |
| SCQ-Total | 20.3±6.04 | 19.23±7.07 | 0.23  (0.6352) | 20±6.76 | 19.26±7.11 | 0.08  (0.7708) | 21.5±2.12 | 18.96±6.84 | 0.27  (0.6070) |

Note: ADI-R = Autism Diagnostic Interview-Revised; SCQ = Social Communication Questionnaire; ADI-R-SOC = Qualitative Abnormalities in Reciprocal Social Interaction of the ADI-R; ADI-R COM = Qualitative Abnormalities in Communication (Verbal) of the ADI-R; ADI-R BEV = Restricted, Repetitive, and Stereotyped Patterns of Behavior of the ADI-R; SCQ-SOC = Social Interaction Subscale of the SCQ ; SCQ –BEV = Repetitive Behavior Subscale; SCQ-COM = Communication Subscale; SCQ-Total = total score of the SCQ. Yes*: indicates patients with rare missense mutations.
